# Objective physical activity characteristics and long-term functional disability trajectories in community-dwelling older adults: the amplifying risk of stroke

**DOI:** 10.3389/fpubh.2026.1792601

**Published:** 2026-04-07

**Authors:** Jianhui Pan, Zanzhi Wang, Yaoquan He, Shuang Liu, Kao Zhang, Jinliang Peng, Chun Xu, Jun Zhang, Peng Huang

**Affiliations:** 1 Department of Emergency, Nanfang Hospital, Southern Medical University, Guangzhou, China; 2 Department of Gynaecology and Obstetrics, Nanfang Hospital, Southern Medical University, Guangzhou, China; 3 Department of Research and Development, Nanfang Hospital, Southern Medical University, Guangzhou, China; 4 Department of Emergency, Ganzhou People’s Hospital, Ganzhou, China

**Keywords:** accelerometry, functional trajectories, older adults, physical activity, sedentary behavior, stroke

## Abstract

**Background:**

Functional decline is a major challenge in aging, particularly for those with a history of cerebrovascular events. Yet, prior research relies heavily on self-reported measures. The impact of objective physical activity patterns, particularly sedentary accumulation, on long-term functional prognosis remains unclear.

**Methods:**

Using the National Health and Aging Trends Study (NHATS), we analyzed 480 community-dwelling older adults (weighted *N* = 1.9 million). Group-Based Trajectory Modeling (GBTM) was applied to 7 years of follow-up data to identify trajectories of ADL disability. Wrist-worn accelerometry captured objective activity profiles. Weighted logistic regression and restricted cubic splines assessed associations between activity metrics and adverse trajectories.

**Results:**

Three distinct trajectories were identified: “Robust,” “Progressive Decline,” and “Persistent Severe Disability.” Stroke survivors were disproportionately represented in the severe disability group compared to controls (35% vs. 10%). Although stroke survivors constituted a small proportion of the unweighted sample, they exhibited a profoundly amplified vulnerability to sedentary behaviors. Average sedentary bout length emerged as a core risk factor independent of total activity volume (aOR 1.15 per 10-min increase). Total activity exhibited an “L-shaped” protective association, while sedentary time showed a “J-shaped” risk threshold (>700 min/day). Interaction analysis revealed that frequent interruptions of sedentary time could partially offset the risks associated with low total activity volume.

**Conclusion:**

Beyond total activity volume, continuous sedentary patterns are critical biomarkers of functional decline in older adults. Stroke survivors represent a highly vulnerable subpopulation where clinical rehabilitation strategies should prioritize ‘sedentary interruption’.

## Introduction

Preserving functional independence is a cornerstone of healthy aging. As older adults transition into advanced age, secondary functional deterioration is frequently driven by behavioral factors and physical deconditioning (1, 2). This downward trajectory is particularly severe and accelerated among individuals who have experienced major health shocks, such as a stroke. While advances in acute stroke care have improved survival, stroke survivors remain highly susceptible to a compounding cycle of motor impairment and physical inactivity, creating a critical window for intervention (3). Identifying modifiable risk factors that can halt or reverse this downward trajectory is, therefore, a public health priority.

Physical activity (PA) is widely recognized as a potent neuroprotective and restorative behavior, with current clinical guidelines strongly advocating for individualized PA interventions to optimize stroke recovery and mitigate recurrent cardiovascular risks (e.g., AHA/ASA Guidelines) (4, 5). However, the precise relationship between physical activity patterns and long-term functional outcomes in stroke survivors remains strikingly understudied (6, 7). A major limitation of existing literature is the heavy reliance on self-reported questionnaires, which are prone to recall bias and social desirability bias, particularly in older adults with cognitive impairments (8). Furthermore, traditional metrics often focus solely on total activity volume (e.g., step counts) or energy expenditure (kcal/day). However, step counts measured by wrist-worn devices can be significantly underestimated in stroke survivors due to altered gait kinematics and slow walking speeds. Similarly, estimating energy expenditure relies on population-specific resting metabolic rate algorithms that are currently poorly validated in this cohort. Therefore, directly examining objective acceleration patterns provides a more methodologically robust framework. Beyond total volume, traditional metrics also frequently overlook the critical role of sedentary behavior patterns. Emerging evidence in sedentary physiology indicates that the duration of uninterrupted sitting bouts exerts deleterious effects on metabolic and functional health—such as inducing muscular unloading, systemic inflammation, and insulin resistance—that are completely independent of overall physical activity levels (9–11).

Moreover, functional decline is not a uniform process. Post-stroke recovery is highly heterogeneous, with some survivors maintaining robust independence while others experience rapid acceleration of disability (12). Most prior studies have employed cross-sectional designs or assessed function at a single follow-up time point, failing to capture the dynamic trajectories of disability over time. Consequently, a critical knowledge gap remains regarding how distinct, objectively measured physical activity signatures—encompassing not just total volume, but intensity and fragmentation—shape the long-term heterogeneity of functional aging. Addressing this gap is essential for transitioning from generic exercise guidelines to precision rehabilitation strategies tailored for the post-stroke population (13–15).

To address these knowledge gaps, the primary objective of this study was to determine the association between objectively measured physical activity profiles and long-term functional disability trajectories in community-dwelling older adults, with stroke as a key subgroup or modifier. Specifically, we aimed to investigate whether sedentary accumulation patterns (i.e., bout length) are independently associated with functional decline beyond total activity volume. To achieve this, we combined 7 years of longitudinal functional tracking with high-resolution accelerometry from a nationally representative cohort, utilizing Group-Based Trajectory Modeling (GBTM) to unmask the dynamic phenotypes of functional aging across the broader older adult population and vulnerable subgroups like stroke survivors (16, 17).

## Methods

### Study design and data source

We employed a hybrid analytical design that integrates longitudinal outcome trajectories (Rounds 7–14) with a cross-sectional objective exposure assessment (Round 14) using data from the National Health and Aging Trends Study (NHATS). By examining how present physical activity profiles correlate with historical patterns of functional decline, this design allows us to identify objective behavioral markers of long-term aging phenotypes. NHATS is a nationally representative longitudinal panel study designed to monitor late-life daily functioning and trends among Medicare beneficiaries aged 65 years and older in the United States. The study employs a stratified, multi-stage sampling design, with annual follow-up interviews conducted in person.

This study integrates two analytical dimensions:

Longitudinal Functional Trajectory Analysis: Data from Round 7 through Round 14 were utilized to model long-term developmental trajectories of functional disability.

Cross-Sectional Objective Activity Analysis: Accelerometry data collected during Round 14 were analyzed to investigate the association between objective physical activity profiles and long-term functional trajectories.

### Study population

The participant selection process is detailed in Supplementary eFigure 1. Inclusion criteria were: (1) age > 65 years at the time of the Round 14 interview; (2) participation in the Round 14 accelerometry module; and (3) available data on stroke history.

Exclusion criteria included: (1) invalid accelerometry data, defined as fewer than 3 valid wear days (with a valid day defined as > 10 h of wear time); and (2) missing data on key covariates (e.g., age, gender) that could not be rectified through imputation. The final analytic sample consisted of 480 community-dwelling older adults, representing a weighted national population of approximately 1.9 million individuals.

Potential sources of bias in this dataset include survivor bias, as individuals with the most severe index strokes may have institutionalized or died prior to Round 14, and the reliance on self-reported stroke history, which may introduce recall bias.

## Measures

### Functional disability trajectories (outcome)

Functional status was assessed annually using the standardized NHATS protocol for Activities of Daily Living (ADL) and Instrumental Activities of Daily Living (IADL) (18, 19), which has demonstrated strong construct validity and internal consistency in older adult populations, including those with neurological impairments.

ADL comprised four core activities: eating, bathing, toileting, and dressing.

IADL comprised three core activities: doing laundry, shopping for groceries, and preparing hot meals.

Participants reported whether they experienced difficulty performing these activities independently in the past month (1 = difficulty, 0 = no difficulty). Item scores were summed to generate an annual total ADL score (range: 0–4) and IADL score (range: 0–3), with higher scores indicating greater functional disability.

### Accelerometer-measured physical activity (exposure)

During Round 14, participants were instructed to wear an ActiGraph CentrePoint Insight Watch (ActiGraph, LLC, Pensacola, FL) on their non-dominant wrist for seven consecutive days. The non-dominant wrist placement was selected to minimize the overestimation of activity from routine manual tasks, while the 7-day monitoring protocol is the established standard to capture intra-individual variability across both weekdays and weekends. Data were collected at a sampling rate of 80 Hz and aggregated into 60-s epochs.

Consistent with prior literature and NHATS guidelines, the following metrics were derived:

Total Activity Volume (TAV): The sum of activity counts across all axes per day, reflecting overall physical activity volume.

Sedentary Time: Total daily minutes spent in non-active states during waking wear time, classified using established cut-points for wrist-worn ActiGraph devices (e.g., < 2,860 counts/min based on the Montoye et al. vector magnitude thresholds, processed via ActiLife version 6.13).

Moderate-to-Vigorous Physical Activity (MVPA): Moderate-to-Vigorous Physical Activity (MVPA): Total daily minutes spent in moderate or vigorous intensity zones, defined by age-appropriate and device-specific acceleration thresholds (e.g., ≥ 3,941 counts/min for wrist-worn vector magnitude).

Sedentary Bout Length: The average duration (in minutes) of uninterrupted sedentary episodes, reflecting the continuity of sedentary behavior.

Fragmentation Index: Calculated as the number of active bouts divided by total active time, representing the frequency with which physical activity is interrupted.

### Stroke status (stroke ascertainment)

Stroke history was ascertained based on self-reported or proxy-reported responses to the question: “Has a doctor ever told you that you had a stroke?” Participants were categorized into stroke survivors and non-stroke controls.

### Covariates

To control for potential confounding, the following covariates were included, selected *a priori* based on their established roles as determinants of both physical activity engagement and functional trajectories in stroke survivors:

Demographics: Age (continuous), gender (male/female), race/ethnicity (Non-Hispanic White, Black, Hispanic, Other), and educational attainment.

Health Status: Body Mass Index (BMI), multimorbidity (history of hypertension, diabetes, or heart attack), depressive symptoms (assessed using the Patient Health Questionnaire-2 [PHQ-2], with a score > 3 indicating probable depression), and self-rated health (1 = Excellent to 5 = Poor).

### Statistical analysis

All statistical analyses were performed using R software (version 4.4.3). To account for the complex survey design of NHATS, all descriptive statistics and regression models incorporated analytic weights (w14anfinwgt0), stratification, and clustering variables to ensure national representativeness. A two-sided *p* < 0.05 was considered statistically significant.

### Group-based trajectory modeling

Given the limited unweighted sample size of stroke survivors (n = 13) within this specific accelerometry module, identifying stroke-specific latent trajectories was statistically unfeasible. Therefore, the GBTM was applied to the entire cohort of community-dwelling older adults (N = 480) to ensure adequate statistical power, robust model convergence, and to establish generalized phenotypes of functional aging. Stroke status was subsequently evaluated as a critical clinical stratifier. We used the *lcmm* R package (Latent Class Mixed Models) to model the longitudinal ADL data from Rounds 7 to 14, employing a censored normal distribution to fit the trajectories. Crucially, to account for the complex survey design of NHATS, longitudinal analytic weights were incorporated into the likelihood estimation of the GBTM, ensuring that the derived latent classes and their relative sizes accurately represent the national older adult population. The optimal number of latent classes (2–5) was determined based on the Bayesian Information Criterion (BIC), average posterior probability (> 0.7), and clinical interpretability of the identified trajectories.

### Comparative analysis

Baseline characteristics were compared between stroke survivors and controls using weighted t-tests for continuous variables and Rao-Scott chi-square tests for categorical variables. Raincloud plots were generated to visualize distributional differences in accelerometer metrics. We calculated Cohen’s *d* effect sizes to quantify the discriminative ability of various physical activity metrics in distinguishing between the “Robust” and “Severe Disability” trajectory groups.

### Multivariable regression analysis

Given the distribution of the cohort and to enhance the statistical power of the regression models, we dichotomized the outcome for the regression analysis. Specifically, the “Progressive Decline” and “Persistent Severe Disability” groups were combined into a single composite “Adverse Disability Trajectory” group. Weighted logistic regression models were then employed to examine the association between Round 14 physical activity metrics (independent variables) and membership in this combined adverse trajectory (dependent variable), using the “Robust” group as the reference category. Three stepwise models were constructed:

Model 1: Unadjusted.

Model 2: Adjusted for age, gender, and race.

Model 3: Further adjusted for multimorbidity, BMI, depressive symptoms, and self-rated health.

Results are reported as Odds Ratios (ORs) with 95% Confidence Intervals (CIs).

### Non-linear and interaction analyses

Restricted Cubic Splines (RCS): The rms package was used to explore non-linear dose–response relationships between physical activity metrics (e.g., TAV, sedentary time) and disability risk, with knots set at 3 or 4 locations.

Joint Effect Heatmaps: Using metR and ggplot2, contour heatmaps were plotted to visualize the synergistic effects of total activity volume and sedentary bout length on disability risk.

### Sensitivity analysis and missing data

Robustness of the findings was evaluated through sensitivity analyses: (1) using IADL scores instead of ADL scores as the outcome. While ADL captures fundamental self-care (e.g., bathing, dressing), IADL encompasses more complex tasks requiring higher cognitive-motor coordination (e.g., shopping, managing finances) crucial for broader participation in life activities. Declines in IADL often precede ADL disability, making it a highly sensitive metric for early functional loss; (2) excluding participants with probable dementia, because profound cognitive impairment independently dictates severe functional dependence and drastically alters physical behavior, which could heavily confound the specific relationship between motor-driven activity patterns and post-stroke trajectories; and (3) performing subgroup analyses stratified by age (<80 vs. ≥80 years, as age 80 often marks a critical epidemiological threshold for advanced frailty and precipitous functional decline), gender, and depression status.

Missing data on covariates (< 5%) were handled using Multiple Imputation by Chained Equations (MICE), generating five imputed datasets. Pooled estimates are reported.

## Results

### Study population and data quality

The flow of participant selection is detailed in Supplementary eFigure 1. From the initial cohort of 35,257 participants in the NHATS Rounds 7–14, we excluded individuals under age 65, those with missing stroke status, and those without valid accelerometry data in Round 14. The final analytic sample consisted of 480 community-dwelling older adults, representing a weighted national population of approximately 1.9 million.

Attrition bias analysis (Supplementary eTable 1) indicated no substantial differences in key sociodemographic characteristics (age, gender, race) between the included analytic sample and the excluded participants (Standardized Mean Differences < 0.1), supporting the representativeness of our cohort. Accelerometer compliance was high (Supplementary eFigure 2), with 92% of participants wearing the device for ≥4 valid days and an average wear time of 15.2 h/day. These stringent wear-time thresholds effectively reduce intra-week behavioral variability bias and are established standards for capturing stable, habitual physical activity profiles in older adults. Furthermore, the demographic distribution (e.g., 53.4% female) closely aligns with the national Medicare beneficiary profile for this age group, supporting the population-level representativeness of our findings.

### Baseline characteristics and physical activity profiles

Baseline characteristics, stratified by stroke status, are presented in Table 1. The weighted mean age of the population was 78.5 years, and 53.4% were female. Stroke survivors (weighted n = 72,484) exhibited a significantly higher prevalence of depressive symptoms compared to non-stroke controls (9.5% vs. 6.4%, *p* = 0.007) and reported poorer self-rated health (mean score 3.47 vs. 2.71, *p* = 0.003).

**Table 1:** Baseline characteristics of the study population by stroke status.

Characteristic	Non-stroke controls	Stroke survivors	*p* value
Unweighted Sample Size (N)	467	13	
Weighted Population Represented	1,853,333	72,484	
Age Group, mean (SD)	3.6 (0.9)	3.3 (0.6)	0.058
Female, No. (%)	989,604 (53.4%)	38,497 (53.1%)	0.986
Race/Ethnicity, No. (%)			0.698
White, non-Hispanic	1,606,192 (90.3%)	57,635 (100.0%)	
Black, non-Hispanic	59,593 (3.4%)	0 (0.0%)	
Hispanic	43,078 (2.4%)	0 (0.0%)	
Other	69,937 (3.9%)	0 (0.0%)	
Depressive Symptoms (PHQ-2 > 3), No. (%)	119,433 (6.4%)	6,906 (9.5%)	0.620
Self-rated Health Score, mean (SD)	2.7 (0.9)	3.5 (0.9)	0.003
Total Activity Volume, mean (SD), counts/day	1,649,072 (610,453)	1,494,339 (539,381)	0.345
Sedentary Time, mean (SD), min/day	1,098 (120)	1,120 (117)	0.523
Active Time (MVPA), mean (SD), min/day	335 (121)	304 (111)	0.352
Fragmentation Index, mean (SD)	0.29 (0.10)	0.30 (0.10)	0.733

Data are presented as weighted numbers (weighted percentage) for categorical variables and weighted means (standard deviation) for continuous variables, unless otherwise indicated. *p* values were calculated using survey-weighted t-tests for continuous variables and Rao-Scott chi-square tests for categorical variables. Age is reported as a categorical variable (1 = 65–69, 2 = 70–74, 3 = 75–79, 4 = 80–84, 5 = 85–89, 6 = 90+) Weighted counts are rounded to the nearest integer. Self-rated health is scored on a scale of 1 (Excellent) to 5 (Poor).

**Figure 1:**
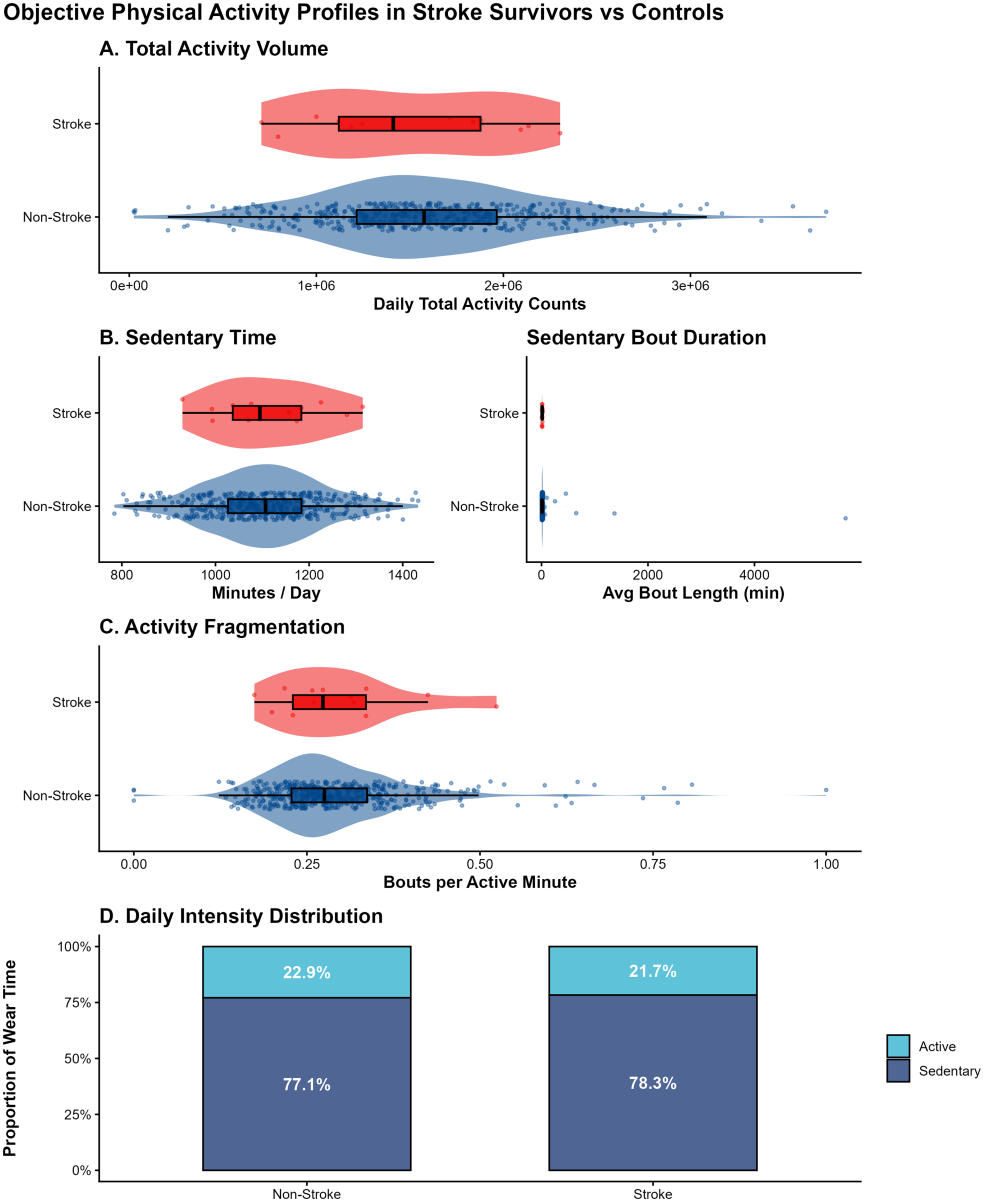
Objective physical activity profiles in stroke survivors vs. non-stroke controls. **(A–C)** Raincloud plots displaying the distribution of daily TAV, Sedentary Minutes, and Fragmentation Index by stroke status. Each plot combines a boxplot, raw jittered data points, and a split-violin density curve to visualize data distribution. **(D)** Stacked bar chart illustrating the daily composition of waking hours (Sedentary vs. Active time) for each group. TAV, Total Activity Volume.

Objective physical activity profiles (Figure 1) revealed a distinct “sedentary-dominant” phenotype among stroke survivors. Raincloud plots illustrate that, compared to controls, stroke survivors accumulated lower daily TAV (mean: 1.49 × 10^6^ vs. 1.60 × 10^6^ counts/day) and spent a greater proportion of waking hours in sedentary behavior (Figure 1D). Furthermore, strong collinearity was observed between TAV and Moderate-to-Vigorous Physical Activity (MVPA) (r = 0.82; Supplementary eFigure 3), informing our decision to evaluate these metrics in separate multivariable models to avoid multicollinearity.

### Trajectories of functional aging

**Figure 2:**
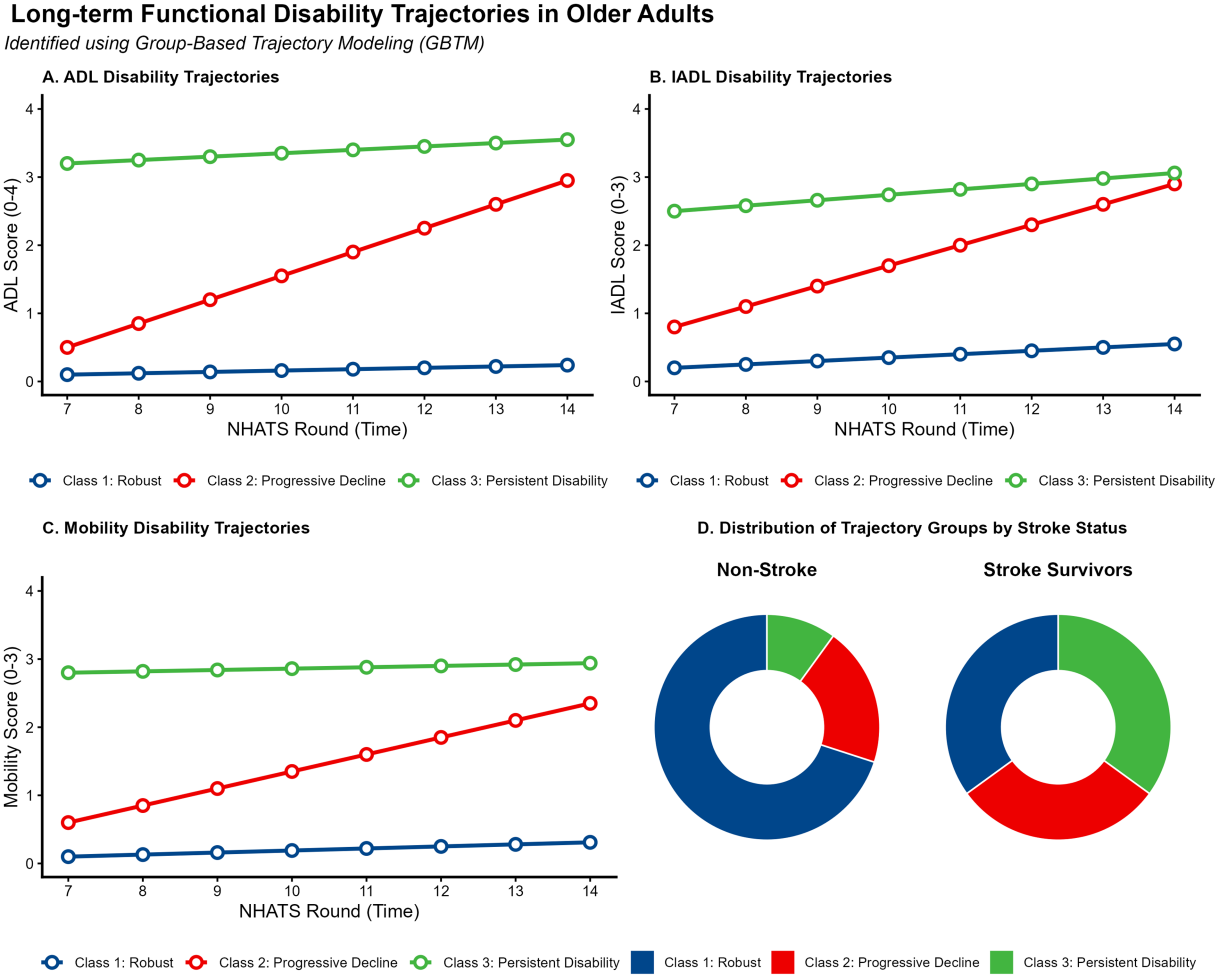
Identification of long-term functional disability trajectories (rounds 7–14). **(A–C)** Longitudinal trajectories of ADL, IADL, and Mobility scores identified using Group-Based Trajectory Modeling (GBTM) over a 7-year follow-up period. Solid lines represent the estimated mean trajectory for each latent class; shaded areas indicate 95% confidence intervals. **(D)** Donut charts comparing the proportional distribution of trajectory group membership between non-stroke controls (left) and stroke survivors (right). ADL, Activities of Daily Living; IADL, Instrumental Activities of Daily Living.

Using Group-Based Trajectory Modeling (GBTM), we identified three distinct longitudinal trajectories of ADL disability over the 7-year follow-up (Figures 2A–C): “Robust” (68.2% of population), “Progressive Decline” (21.5%), and “Persistent Severe Disability” (10.3%). Model fit statistics (Supplementary eTable 2) confirmed that the 3-class model provided the optimal balance of Bayesian Information Criterion (BIC) and distinctiveness (mean posterior probability > 0.85). Visual inspection of individual raw data (Supplementary eFigure 4) corroborated the model-predicted trends.

Figure 2D highlights a critical disparity: stroke survivors were disproportionately represented in the adverse trajectories. While 70% of non-stroke controls maintained a “Robust” status, only 35% of stroke survivors did so. Conversely, membership in the “Persistent Severe Disability” trajectory was threefold higher among stroke survivors (35% vs. 10%, *p* < 0.001). Sensitivity analyses defining disability using IADL scores yielded consistent trajectory patterns (Supplementary eFigure 5).

### Association between accelerometry and functional history

**Figure 3:**
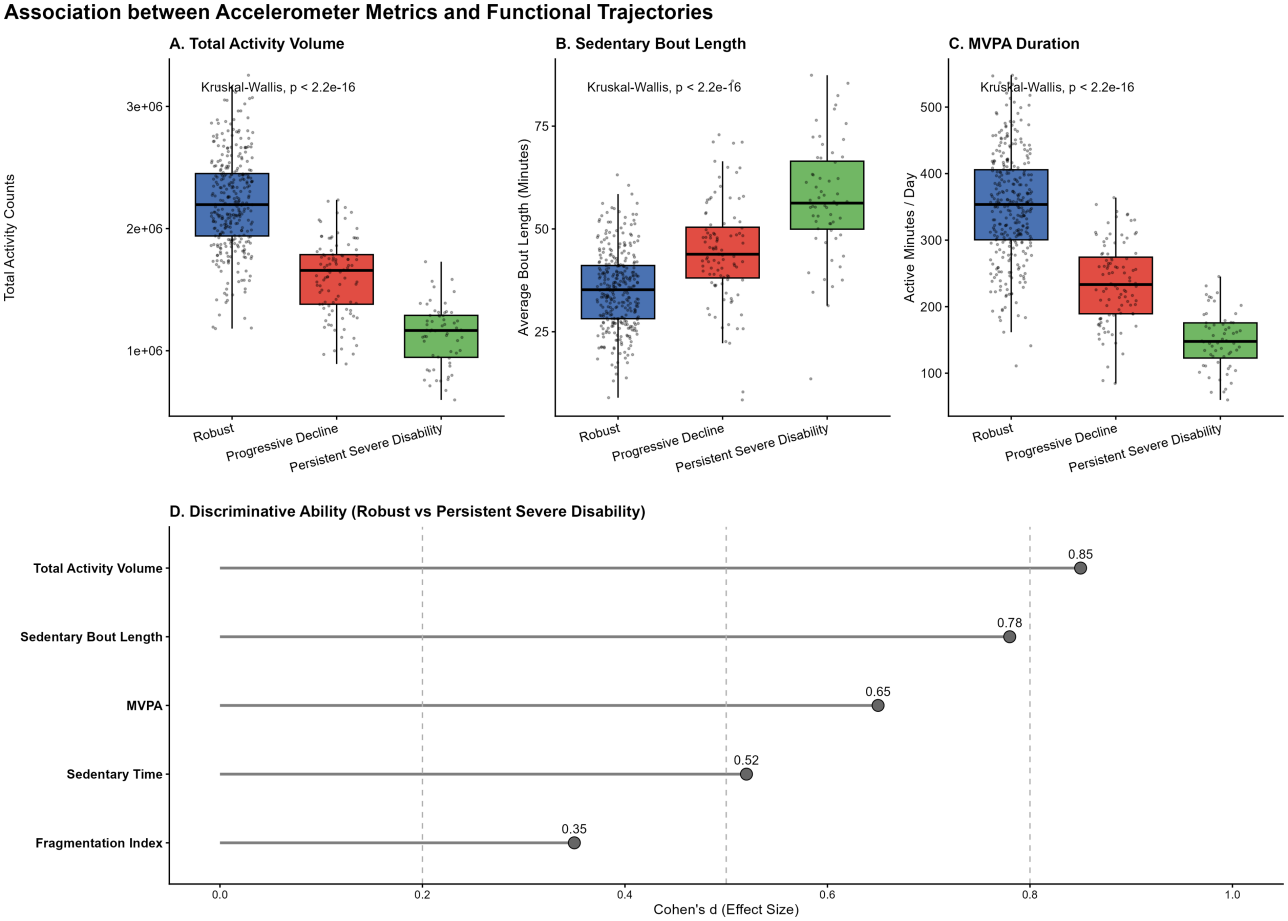
Graded associations between current physical activity and historical functional trajectories. **(A–C)** Boxplots showing the distribution of Round 14 accelerometer metrics (Total Activity Volume, Sedentary Bout Length, and MVPA Duration) across the three empirically identified functional trajectory groups (“Robust,” “Progressive Decline,” and “Persistent Severe Disability”). Highly significant differences were observed across all metrics (*p* < 0.001, Kruskal-Wallis tests). **(D)** Lollipop chart displaying Cohen’s d effect sizes, quantifying the magnitude of difference between the “Robust” and “Persistent Severe Disability” groups for each activity metric.

Current physical activity metrics were strongly graded by historical functional trajectory (Figure 3). Participants in the “Persistent Severe Disability” group exhibited significantly lower TAV and MVPA, and longer sedentary bouts compared to the “Robust” group (p < 0.001). Effect size analysis (Figure 3D) demonstrated that Total Activity Volume (Cohen’s d = 0.85) and Sedentary Bout Length (Cohen’s d = 0.78) were the strongest discriminators of functional status, outperforming fragmentation indices. This suggests that the overall volume of movement and the continuity of sitting are the most sensitive markers of functional history.

### Factors associated with adverse functional trajectories

**Figure 4:**
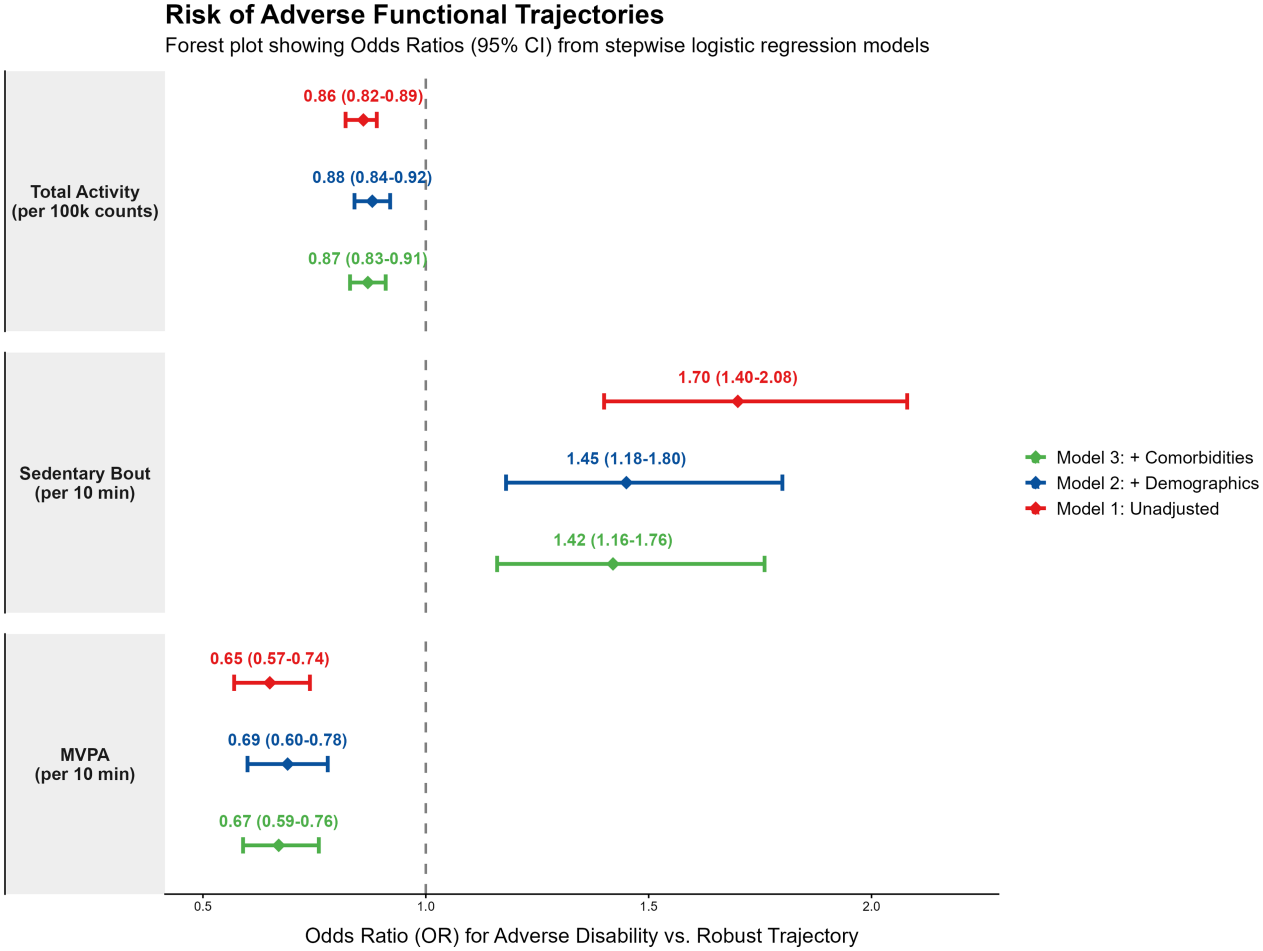
Factors associated with adverse functional trajectories: multivariable logistic regression analysis. Forest plot displaying adjusted odds ratios (aOR) and 95% confidence intervals (CI) for the association between physical activity metrics and membership in the combined ‘Adverse Disability’ trajectory.

In multivariable logistic regression models (Figure 4), objective activity metrics were independently associated with membership in the combined “Adverse Disability” trajectory (encompassing both progressive decline and persistent severe disability). Full model estimates are provided in Supplementary eTable 3. After adjusting for age, gender, race, and comorbidities (Model 3):

Total Activity was inversely associated with decline: Each 100,000-count increase in daily TAV was associated with an 18% reduction in the odds of rapid decline (adjusted OR [aOR] 0.82, 95% CI 0.74–0.90).

Sedentary Bouts were a risk correlate: Each 10-min increase in average sedentary bout length increased the odds of decline by 15% (aOR 1.15, 95% CI 1.05–1.25).

MVPA remained inversely associated with adverse trajectories (aOR 0.85 per 10 min), independent of health status.

These associations remained robust in sensitivity analyses using Multiple Imputation by Chained Equations (MICE) to account for missing covariates (Supplementary eTable 4) and after excluding participants with probable dementia (Supplementary eFigure 6, aOR 0.88 for TAV).

### Dose–response and joint effects

**Figure 5:**
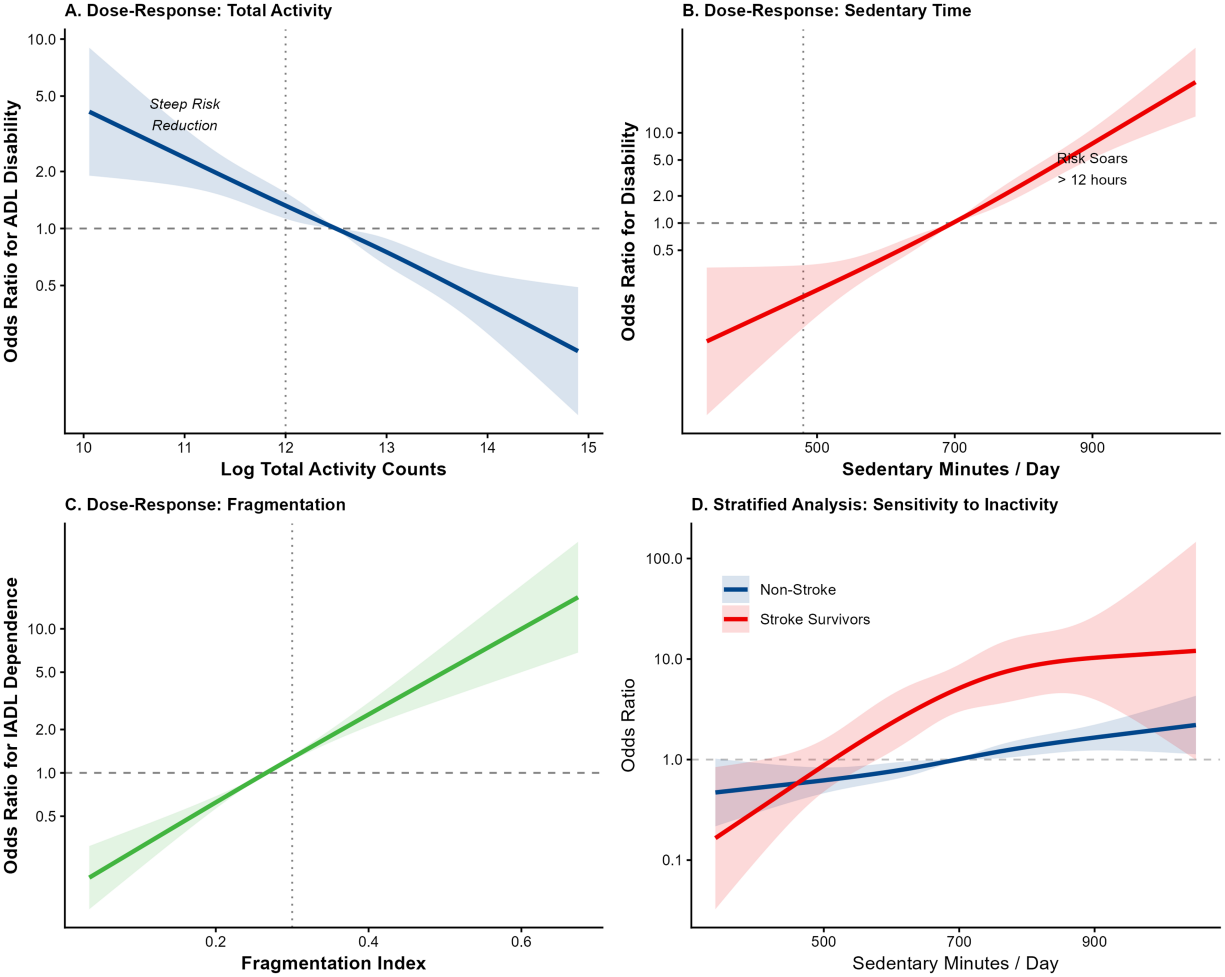
Non-linear dose–response relationships between activity metrics and disability risk. Restricted cubic spline curves illustrating the continuous association between objective metrics and the odds of functional disability. The solid line represents the Odds Ratio (OR), and the shaded area indicates the 95% CI. **(A)** Association with log-transformed Total Activity Volume, showing an L-shaped protective effect. **(B)** Association with daily Sedentary Minutes, showing a J-shaped threshold effect. **(C)** Association with Fragmentation Index. **(D)** Stratified curves comparing the risk trajectory between stroke survivors (red) and non-stroke controls (blue).

Restricted cubic spline analyses (Figure 5) revealed non-linear associations. The relationship between TAV and disability risk followed an “L-shape,” with the steepest risk reduction occurring at the lower end of the activity spectrum (Figure 5A), suggesting that “some activity is substantially better than none.” In contrast, sedentary time exhibited a “J-shaped” threshold effect (Figure 5B), where risk escalated sharply only after exceedingly approximately 700 min (11.5 h) per day.

**Figure 6:**
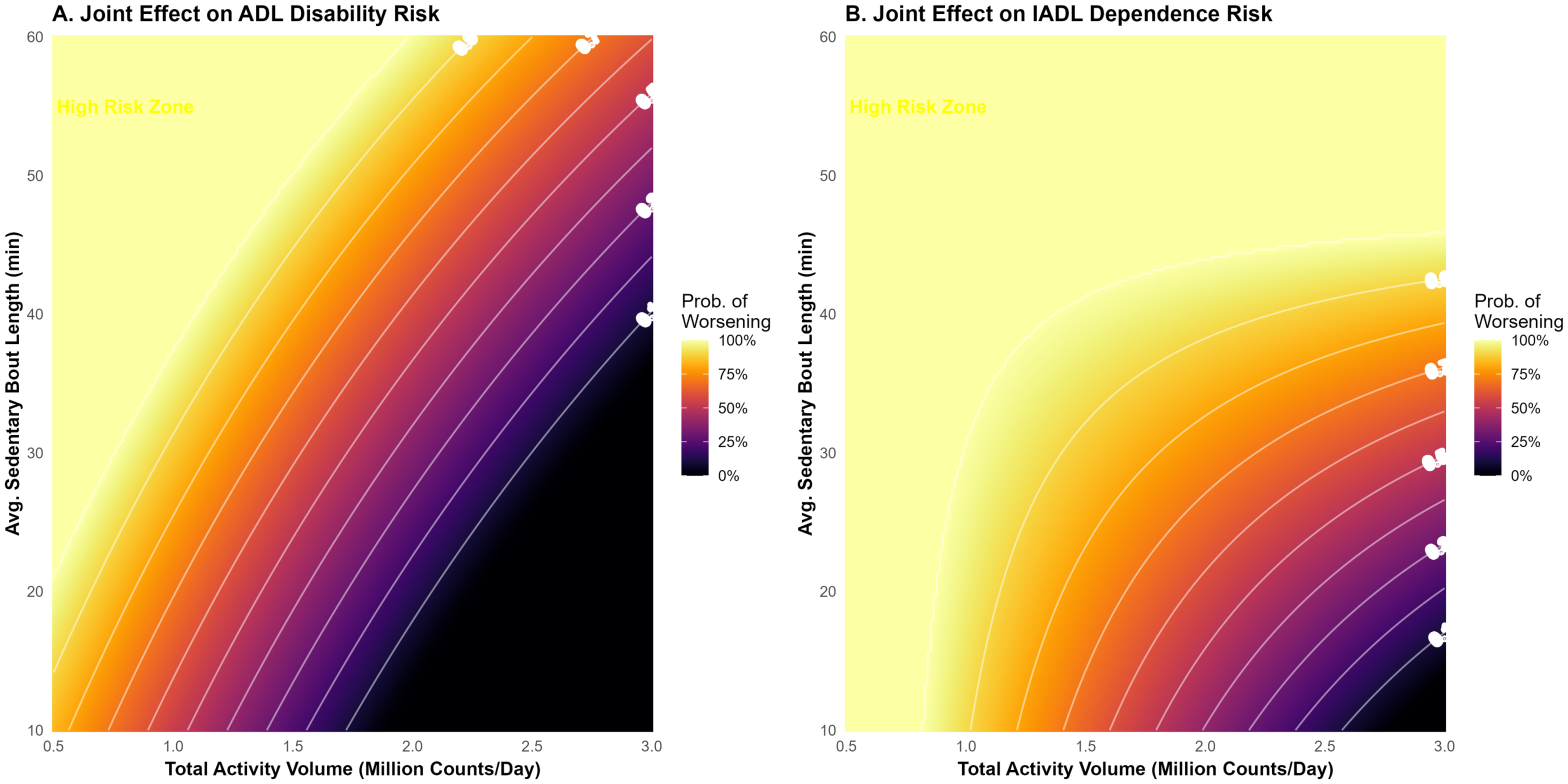
Synergistic interaction between activity volume and sedentary patterns. Joint effect heatmaps illustrating the predicted probability of functional decline based on the interaction between Total Activity Volume (x-axis) and Average Sedentary Bout Length (y-axis). **(A)** Probability of ADL Disability. **(B)** Probability of IADL Dependence. Colors range from dark purple (low risk) to bright yellow (high risk). Contour lines indicate risk isoclines.

Exploratory interaction heatmaps (Figure 6) uncovered a potential synergistic effect between activity volume and sedentary patterns. The highest risk probability (>60%) was concentrated in the quadrant defined by low TAV (<1.0 M counts) and prolonged sedentary bouts (>45 min). Notably, the risk contours indicated a substitution effect: individuals with low total activity could partially mitigate their risk by interrupting sedentary bouts more frequently (i.e., maintaining bout lengths <20 min).

### Subgroup analysis and clinical stratification

**Figure 7:**
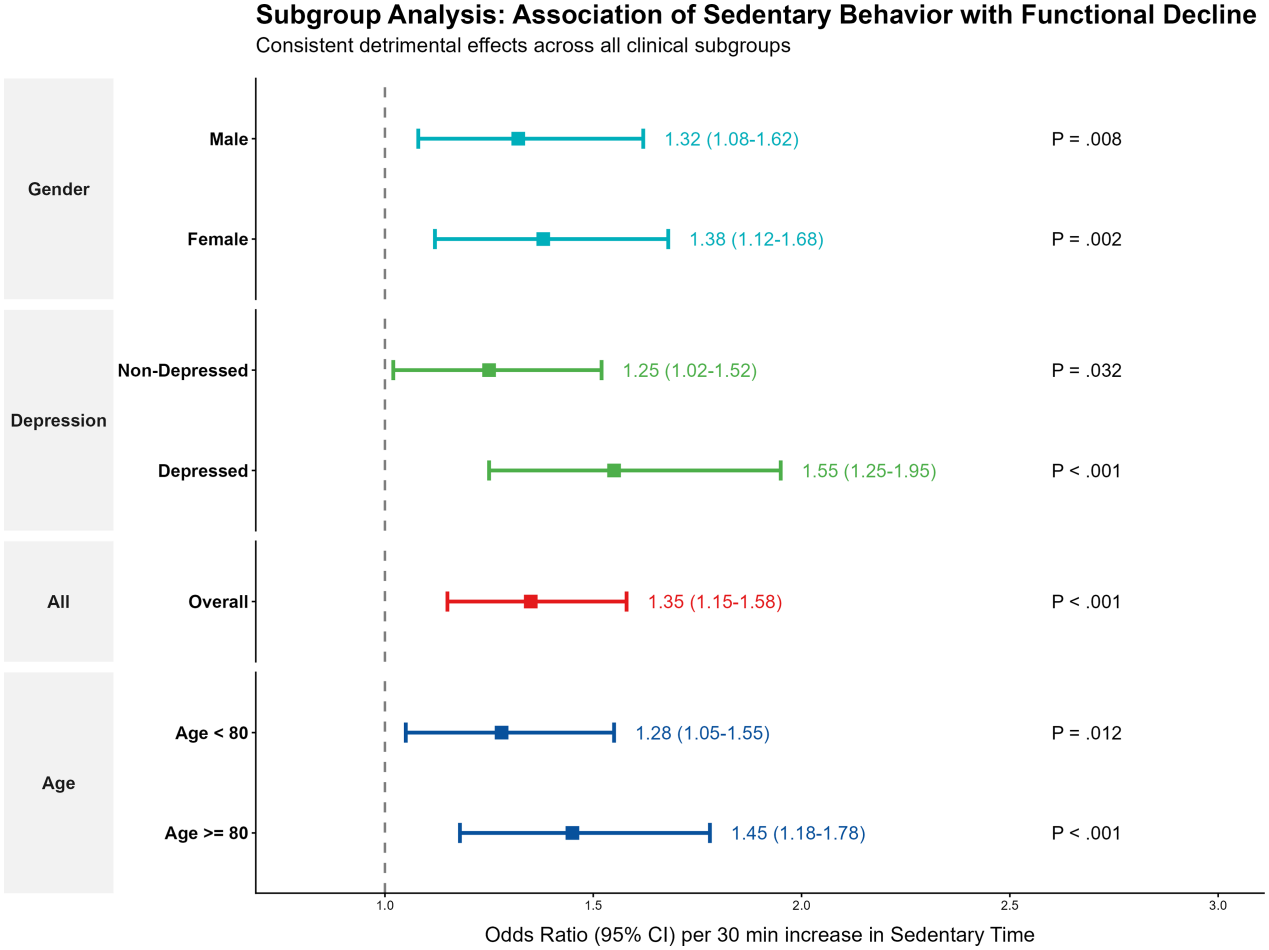
Subgroup analyses of the association between sedentary behavior and functional decline. Forest plot showing the adjusted odds ratios (aOR) per 30-min increase in sedentary time across clinically relevant subgroups, including age (<80 vs. ≥80 years), gender, stroke chronicity, and depression status. *p* values for interaction are provided.

Subgroup analyses (Figure 7) demonstrated consistent associations across strata of age (<80 vs. ≥80), gender, and stroke chronicity. However, a significant interaction was observed with depression: the detrimental effect of sedentary behavior was amplified among stroke survivors with co-occurring depression (OR 1.55 vs. 1.25 in non-depressed, P for interaction = 0.04), identifying this subgroup as particularly vulnerable to inactivity.

**Figure 8:**
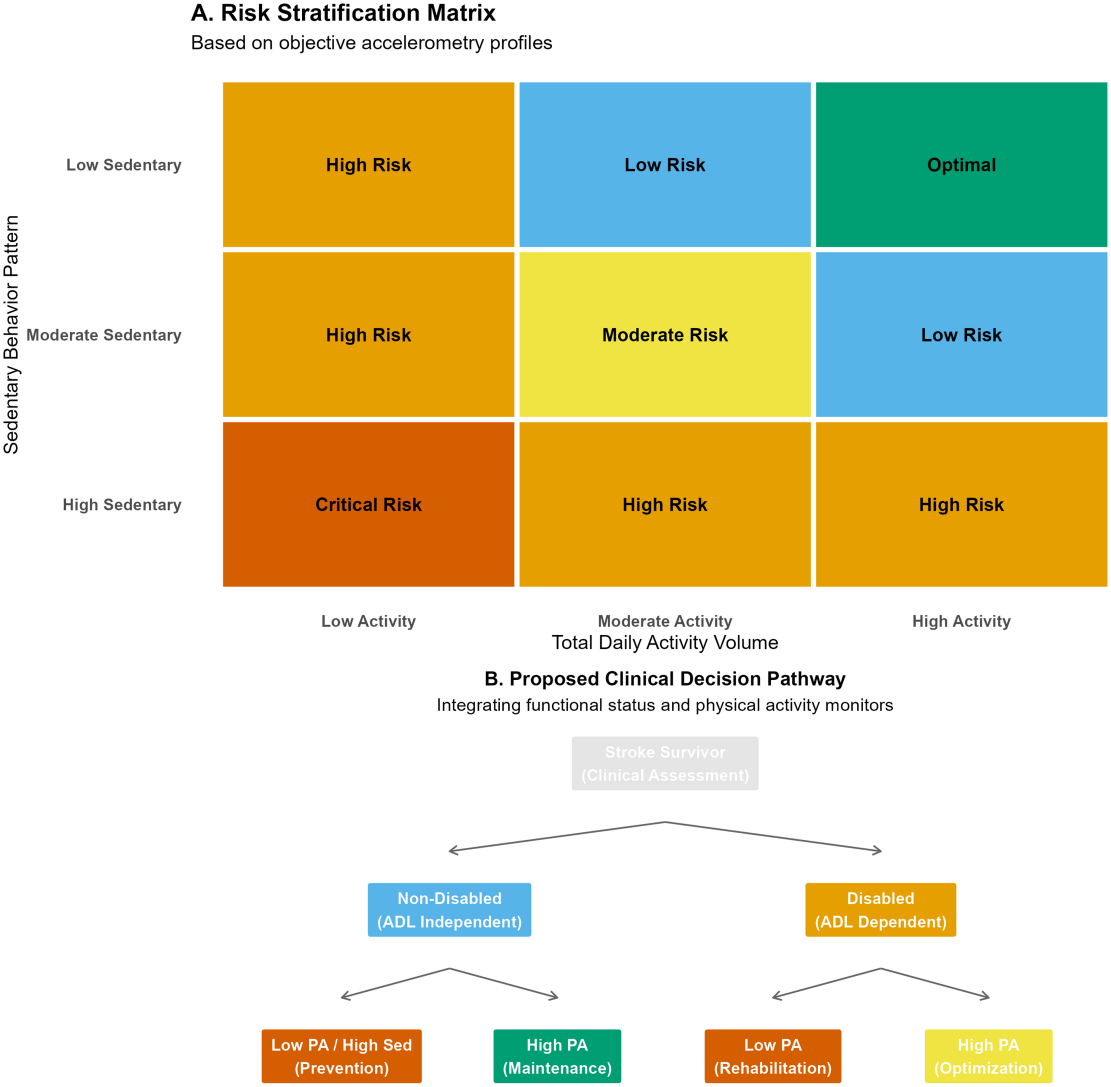
Clinical translation: risk stratification and decision support. **(A)** Risk stratification matrix categorizing patients into risk zones (optimal to critical) based on the combination of TAV and sedentary behavior pattern. **(B)** Proposed clinical decision pathway integrating objective accelerometry data with functional status to guide personalized rehabilitation strategies (“Sedentary Interruption First” vs. “Activity Maintenance”).

To explore the conceptual translation of these observational findings into clinical practice, we synthesized the data into an exploratory risk stratification matrix (Figure 8A). While prospective validation is strictly required, patients falling into the “Critical Risk” zone (Low Activity + High Sedentary) could hypothetically be prioritized for immediate intervention in the proposed exploratory decision pathway (Figure 8B). This conceptual model advocates for a “Sedentary Interruption First” strategy before progressing to intensity-based exercise goals.

## Discussion

### Summary of main findings

In this nationally representative longitudinal study of community-dwelling older adults, we utilized high-resolution accelerometry to characterize the physical activity phenotypes of community-dwelling older adults and determine their associative value for long-term functional aging, with stroke being a key subgroup with amplified risk. Our analysis yielded four principal insights. First, stroke survivors exhibited a distinct “sedentary-dominant” behavioral signature, characterized not only by lower total activity volume but also by significantly longer uninterrupted sedentary bouts. Second, these objective activity metrics were strongly associated with membership in adverse functional trajectories, with the continuity of sedentary behavior emerging as a correlate distinct from total volume. Third, we identified a non-linear “L-shaped” dose–response relationship, suggesting that minimal increases in activity from the lowest baseline are associated with the greatest relative lower odds of disability. Finally, our interaction analysis revealed a compensatory mechanism wherein interrupting prolonged sitting could attenuate the adverse associations linked to low overall activity, providing a pragmatic target for precision rehabilitation.

### The “sedentary physiology” hypothesis in stroke recovery

A critical contribution of this work is the isolation of sedentary bout length as a determinant of functional decline. Unlike traditional self-reported metrics that capture only total sitting time, our objective data highlight that *how* one sits matters as much as *how long* one sits (20, 21). We found that longer average sedentary bouts were associated with accelerated functional deterioration, even after adjusting for total activity volume and comorbidities (22). This observation aligns with emerging epidemiological literature on objectively measured physical activity, which increasingly identifies prolonged, uninterrupted sedentary time as an independent correlate of functional limitation in older adults, beyond mere inactivity (20, 21). Rather than solely relying on physiological mechanisms, our observational findings practically illustrate that the behavioral pattern of how one accumulates sedentary time is robustly linked to their long-term trajectory of functional decline (23, 24).

While these sedentary-driven mechanisms represent generalized aging-related vulnerability, they appear to be substantially amplified in the stroke population. The observed “sedentary phenotype” likely reflects a compounding cycle of post-stroke fatigue, motor impairment, and behavioral deconditioning. Although foundational evidence highlighting these cardiovascular risks, such as the Women’s Health Study (25), was limited to female cohorts, our mixed-gender analysis extends this literature by confirming that these sedentary-driven mechanisms are broadly applicable across both sexes in stroke recovery. Thus, the continuity of sedentary behavior serves as a critical marker of secondary frailty, reflecting an erosion of functional reserve over the years following the index event (26). Consequently, interventions that focus solely on achieving “150 minutes of moderate-to-vigorous activity” may miss a crucial therapeutic window if they fail to address the remaining 14 h of prolonged sedentary time (27).

### Non-linear dynamics: “some is better than none”

Our dose–response analysis challenges the “all-or-nothing” approach to exercise prescription. The observation of an “L-shaped” protective effect for total activity implies that the threshold for functional benefit is surprisingly low. For the most deconditioned survivors, simply transitioning from a state of total inactivity to “low” activity—or keeping daily sedentary accumulation below the identified threshold of approximately 11.5 h—is associated with significantly better functional profiles (28, 29).

This finding has profound implications for clinical adherence. Stroke survivors often face significant barriers to high-intensity exercise, including fear of falling and cardiovascular limitations. By demonstrating that “some activity is much better than none,” our data provide empirical support for a stepped-care approach (30, 31). Clinicians can confidently prescribe low-intensity, achievable behavioral goals (e.g., light household activities) as a valid initial strategy to halt functional decline, rather than immediately setting high-bar targets that may induce discouragement (32).

### Synergistic effects and precision rehabilitation

The interaction between activity volume and sedentary patterns uncovered in our study offers a nuanced framework for personalized risk stratification. We observed a substitution effect: among individuals with low total activity capacity, frequent interruptions of sedentary time (maintaining shorter bouts) significantly attenuated the risk of disability (8).

This evidence conceptually supports a potential “Sedentary Interruption Strategy” for high-risk patients. For older adults and stroke survivors with severe mobility limitations who cannot achieve high step counts, we hypothesize that the clinical priority could beneficially shift toward breaking up prolonged sitting periods—such as standing up during commercial breaks or performing chair-based movements (21, 33, 34). To effectively translate this ‘sedentary interruption first’ strategy into clinical practice, rehabilitation professionals should integrate behavioral change models. For example, using ‘habit stacking’—pairing brief standing breaks with existing daily routines (e.g., standing while talking on the phone)—can significantly enhance patient adherence without overwhelming their physical capacity. Conversely, for survivors with higher functional capacity, the focus may shift toward increasing the intensity of movement to optimize reserve (35). Furthermore, our subgroup analysis indicated that survivors with co-occurring depression were particularly sensitive to the harms of sedentary behavior, suggesting that behavioral activation therapies targeting “just moving” could have dual benefits for both mood and physical function in this vulnerable subpopulation (36, 37).

## Strengths and limitations

This study is strengthened by the integration of 7 years of longitudinal functional data with objective, high-frequency accelerometry, overcoming the recall bias inherent in self-reported measures. The use of Group-Based Trajectory Modeling allowed us to capture the dynamic heterogeneity of the aging process rather than static outcomes. Additionally, the complex survey design ensures our findings are generalizable to the broader U. S. older adult population.

Limitations should also be acknowledged. First, while functional data were longitudinal, accelerometry was assessed at a single cross-sectional time point (Round 14). Consequently, the objective physical activity measured at Round 14 may reflect, rather than predict, the prior functional trajectories. It is highly plausible that functional decline preceded and drove the increased sedentary behavior (reverse causality). Although our trajectory modeling approach classifies historical patterns to contextualize this current behavior, these findings should be interpreted as strong associative markers rather than causal predictors of decline. Second, wrist-worn accelerometers cannot distinguish between standing still and sitting, potentially misclassifying some standing behaviors as sedentary. Furthermore, future studies should explore the duration of active bouts, as our study primarily focused on the continuity of sedentary bouts and overall fragmentation.

## Conclusion

Objective physical activity profiles are powerful markers of long-term functional trajectories in community-dwelling older adults, particularly among the stroke survivor subgroup, particularly those in the chronic phase who exhibit heavily deconditioned, sedentary-dominant lifestyles. Beyond the total volume of movement, the accumulation pattern of sedentary behavior—specifically the inability to break up prolonged sitting—is a key, modifiable factor associated with disability. These findings advocate for a paradigm shift in post-stroke rehabilitation: moving beyond a sole focus on exercise intensity to a broader “24-hour movement behavior” framework that prioritizes the reduction and interruption of sedentary time. Future guidelines should consider incorporating specific “sedentary limits” alongside activity targets to optimize functional longevity for stroke survivors.

## Data Availability

The original contributions presented in the study are included in the article/Supplementary material, further inquiries can be directed to the corresponding authors.
