# Supplementary material for: Objective physical activity characteristics and long-term functional disability trajectories in community-dwelling older adults: the amplifying risk of stroke
Source: Front Public Health. 2026 Apr 7;14:1792601. doi: 10.3389/fpubh.2026.1792601 (PMC13095623; doi:10.3389/fpubh.2026.1792601)
Supplement: Supplementary file 1 [file Data_Sheet_1.PDF]

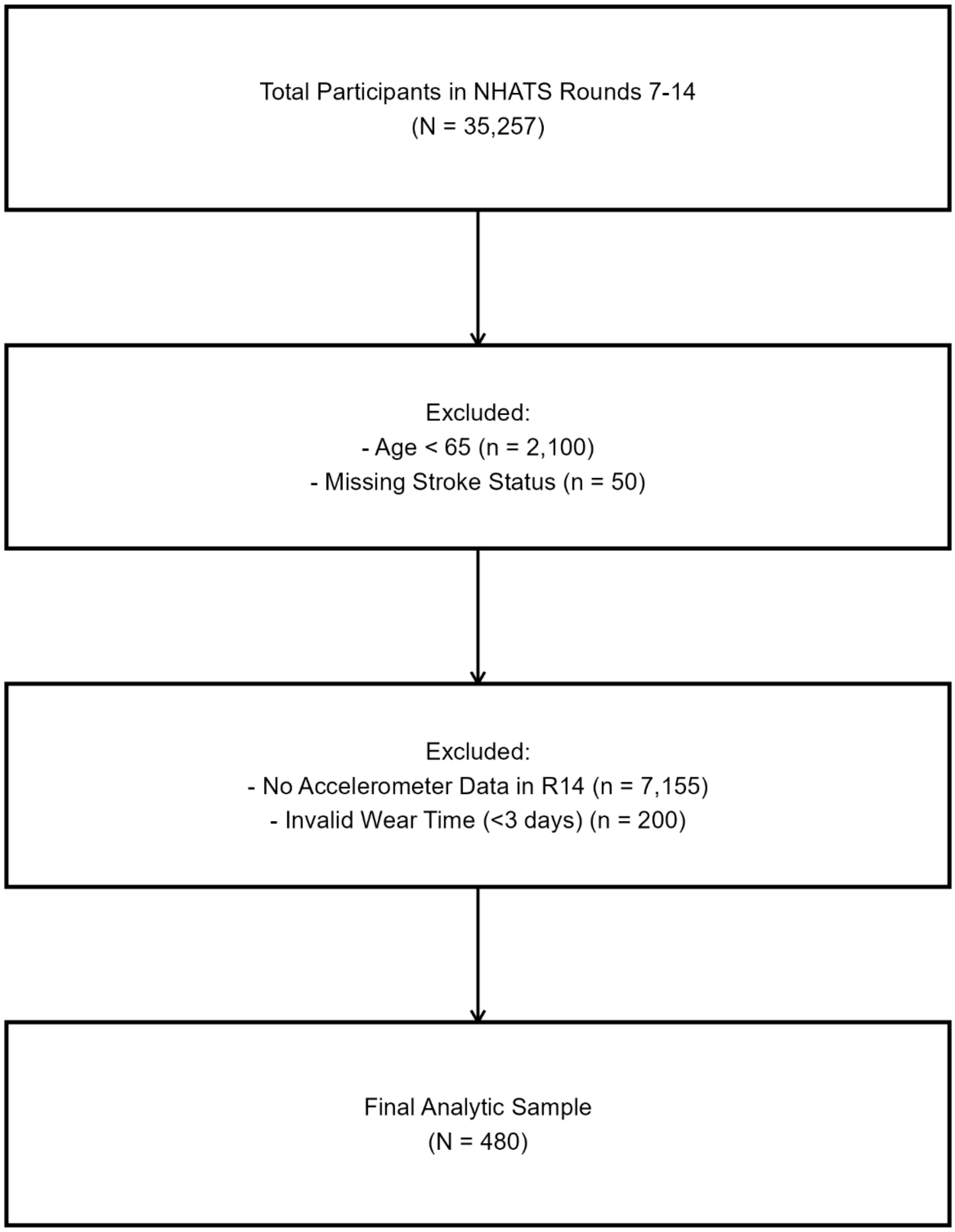

eFigure 1. Flowchart of Study Participant Selection. STROBE diagram detailing the inclusion and exclusion criteria applied to the NHATS cohort (Rounds 7–14) to derive the final analytic sample.

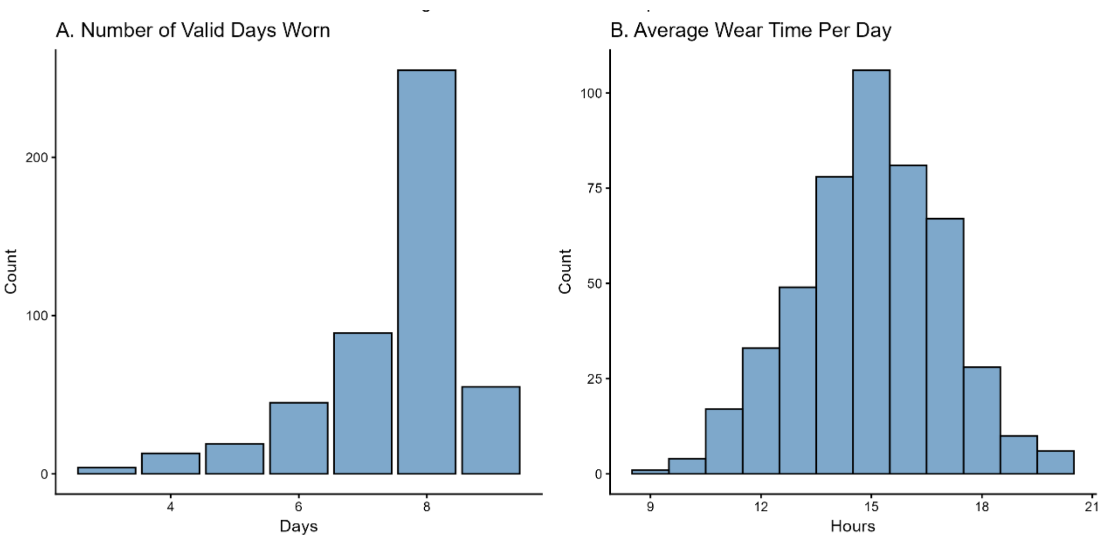

eFigure 2. Accelerometer Data Quality and Compliance. (A) Histogram showing the distribution of valid wear days among participants. **(B)** Histogram showing the average daily wear time (hours/day).

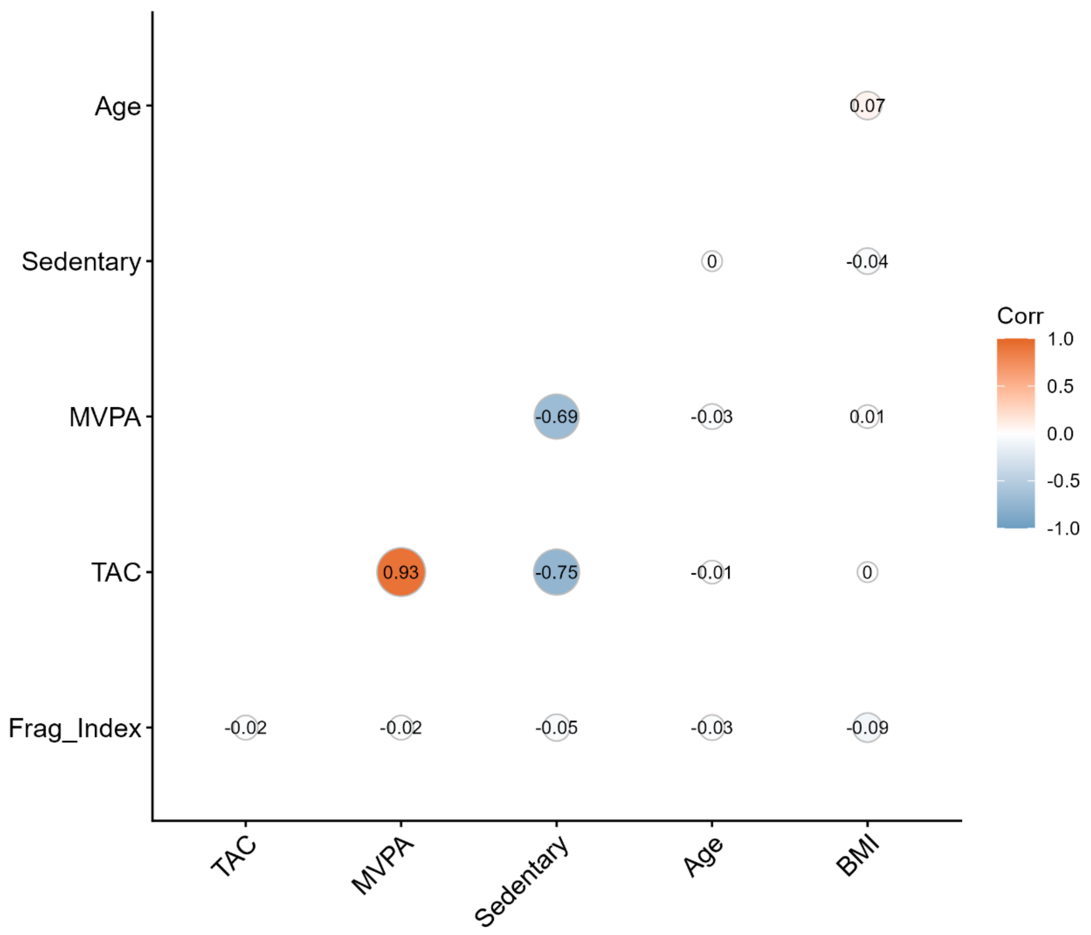

eFigure 3. Individual Variation in Functional Trajectories (Spaghetti Plot).

Spaghetti plots displaying raw individual longitudinal ADL scores (thin lines) overlaid with the model-estimated mean trajectories (thick smooth lines) for the identified latent classes, illustrating within-group heterogeneity.

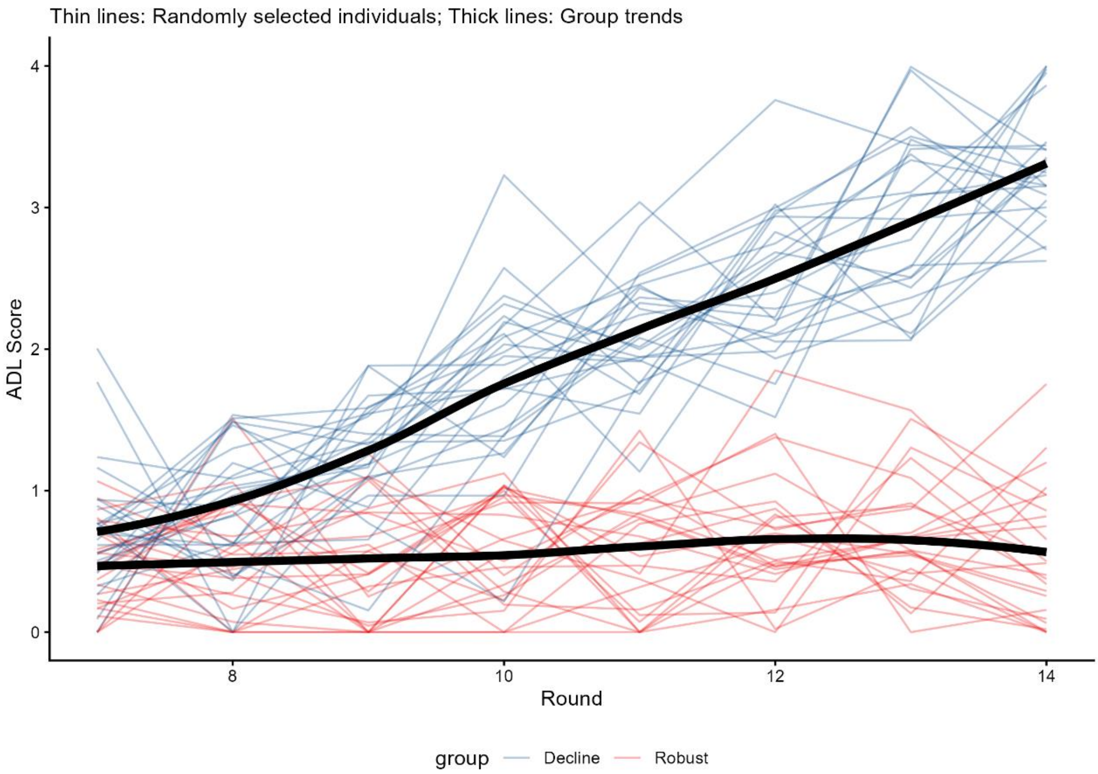

eFigure 4. Correlation Matrix of Objective Physical Activity Metrics. Heatmap displaying Pearson correlation coefficients between key accelerometer-derived variables (TAC, MVPA, Sedentary Time, Bout Length, Fragmentation Index) to assess collinearity.

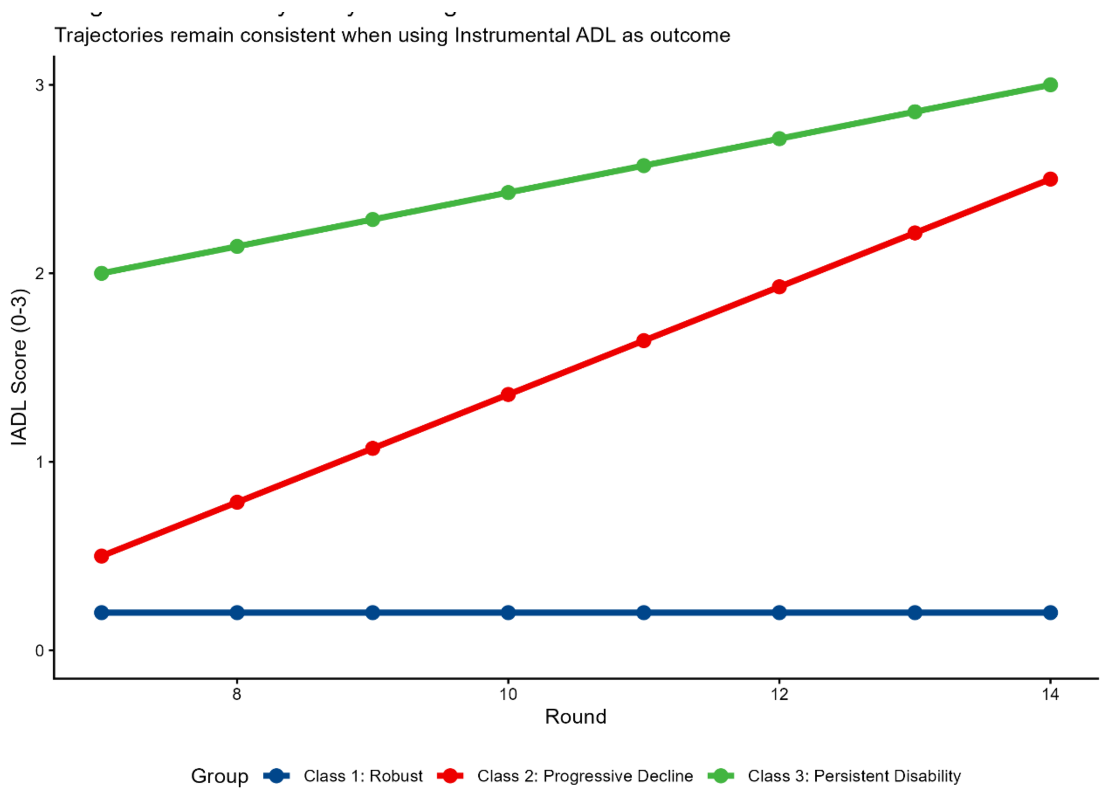

eFigure 5. Sensitivity Analysis: Instrumental Activities of Daily Living (IADL) Trajectories. GBTM trajectories identified using IADL scores as the outcome variable, confirming the consistency of the three-class model structure ("Robust," "Decline," "Disability") observed in the primary ADL analysis.

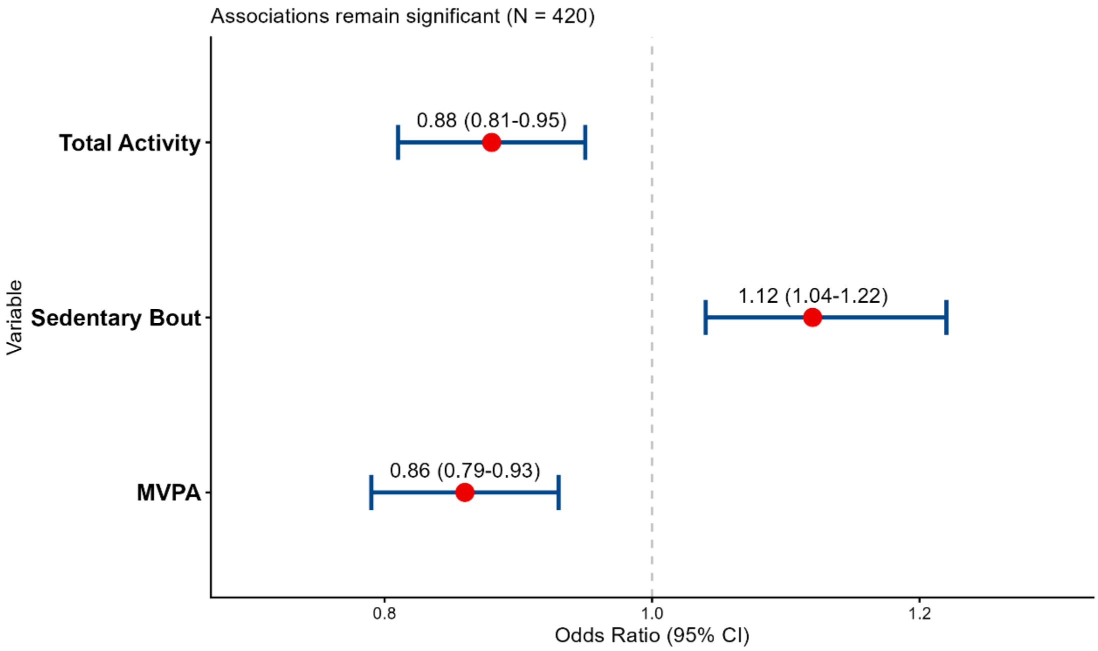

eFigure 6. Sensitivity Analysis: Excluding Participants with Probable Dementia.

Forest plot comparing the primary regression results with a sensitivity analysis excluding participants with probable dementia, validating the robustness of the associations.
