# Supplementary material for: Objective physical activity characteristics and long-term functional disability trajectories in community-dwelling older adults: the amplifying risk of stroke
Source: Front Public Health. 2026 Apr 7;14:1792601. doi: 10.3389/fpubh.2026.1792601 (PMC13095623; doi:10.3389/fpubh.2026.1792601)
Supplement: Supplementary file 2 [file Table_1.DOCX]

**eTable 1. Comparison of Baseline Characteristics Between Included and Excluded Participants**

| Characteristic | Included Analytic Sample (N = 480) | Excluded Participants (N = 7,355) | Standardized Mean Difference (SMD) |
| --- | --- | --- | --- |
| Age, mean (SD), y | 78.5 (7.2) | 79.1 (8.0) | 0.08 |
| Female, No. (%) | 265 (55.2%) | 4,178 (56.8%) | 0.03 |
| Race/Ethnicity, No. (%) |  |  | 0.04 |
| White | 394 (82.1%) | 5,920 (80.5%) |  |
| Black | 16 (3.4%) | 289 (3.9%) |  |
| Hispanic | 19 (4.0%) | 255 (3.5%) |  |
| Other | 51 (10.6%) | 891 (12.1%) |  |
| Stroke History, No. (%) | 13 (2.7%) | 213 (2.9%) | 0.01 |
| Depressive Symptoms (PHQ-2 > 3), No. (%) | 31 (6.4%) | 522 (7.1%) | 0.05 |
| Self-rated Health (Fair/Poor), No. (%) | 90 (18.7%) | 1,412 (19.2%) | 0.02 |

*Note: Data are presented as unweighted numbers (percentage) unless otherwise indicated. SMD: Standardized Mean Difference. An SMD < 0.1 indicates a negligible difference between groups, supporting the representativeness of the analytic sample. Exclusion criteria: Participants were excluded if they were aged <65 years, had missing stroke status, or lacked valid accelerometry data in Round 14 (<3 valid days).*

**eTable 2. Goodness-of-Fit Statistics for Group-Based Trajectory Modeling (GBTM)**

| Number of Classes | BIC | AIC | Average Posterior Probability | Smallest Class Size (%) |
| --- | --- | --- | --- | --- |
| 2 | 12,580.4 | 12,540.1 | 0.92 | 45.2% |
| 3 | 12,450.2 | 12,400.5 | 0.88 | 10.3% |
| 4 | 12,455.6 | 12,395.2 | 0.81 | 4.1% |
| 5 | 12,468.9 | 12,390.1 | 0.76 | 1.8% |

*Note: BIC: Bayesian Information Criterion; AIC: Akaike Information Criterion. Models were fitted using censored normal distribution for ADL scores over Rounds 7–14. The 3-class model was selected as the final model because it minimized the BIC and maintained conceptually distinct trajectory groups with adequate sample sizes (>5%). The three identified classes were labeled as "Robust" (68.2%), "Progressive Decline" (21.5%), and "Persistent Severe Disability" (10.3%).*

**eTable 3. Multivariable Logistic Regression Analysis of Physical Activity Metrics Predicting Rapid Functional Decline**

| Predictor | Model | Odds Ratio (95% CI) | P Value |
| --- | --- | --- | --- |
| Total Activity Volume (TAC) |  |  |  |
| *(per 100,000 counts/day)* | Model 1 | 0.75 (0.68 – 0.82) | <.001 |
|  | Model 2 | 0.78 (0.71 – 0.86) | <.001 |
|  | Model 3 | 0.82 (0.74 – 0.90) | <.001 |
|  |  |  |  |
| MVPA Duration |  |  |  |
| *(per 10 min/day)* | Model 1 | 0.72 (0.65 – 0.80) | <.001 |
|  | Model 2 | 0.79 (0.72 – 0.88) | <.001 |
|  | Model 3 | 0.85 (0.76 – 0.92) | .002 |
|  |  |  |  |
| Sedentary Bout Length |  |  |  |
| *(per 10 min increase)* | Model 1 | 1.22 (1.12 – 1.34) | <.001 |
|  | Model 2 | 1.18 (1.08 – 1.29) | <.001 |
|  | Model 3 | 1.15 (1.05 – 1.25) | .004 |

*Note: The outcome variable is membership in the "Rapid Decline/Persistent Disability" trajectory group (vs. "Robust" group). OR: Odds Ratio; CI: Confidence Interval; TAC: Total Activity Counts; MVPA: Moderate-to-Vigorous Physical Activity. Model 1: Unadjusted. Model 2: Adjusted for Age, Gender, and Race. Model 3: Adjusted for Age, Gender, Race, Hypertension, Diabetes, Heart Attack, Depression, BMI, and Self-rated Health. Statistically significant results (P < .05) in the fully adjusted model are bolded.*

**eTable 4. Sensitivity Analysis: Comparison of Results Using Complete Case Analysis vs. Multiple Imputation**

| Physical Activity Metric | Model Strategy | Adjusted OR (95% CI) | P Value |
| --- | --- | --- | --- |
| Total Activity Volume | Complete Case (N=425) | 0.81 (0.72 – 0.91) | .001 |
| *(per 100,000 counts)* | MICE Imputed (N=480) | 0.82 (0.74 – 0.90) | <.001 |
|  |  |  |  |
| MVPA Duration | Complete Case (N=425) | 0.83 (0.74 – 0.91) | .002 |
| *(per 10 min)* | MICE Imputed (N=480) | 0.85 (0.76 – 0.92) | .002 |
|  |  |  |  |
| Sedentary Bout Length | Complete Case (N=425) | 1.16 (1.04 – 1.28) | .008 |
| *(per 10 min)* | MICE Imputed (N=480) | 1.15 (1.05 – 1.25) | .004 |

*Note: All models are fully adjusted (corresponding to Model 3 in eTable 3). MICE: Multiple Imputation by Chained Equations. We generated 5 imputed datasets to handle missing data on covariates (e.g., BMI, Depression scores). The pooled estimates from MICE are reported. The similarity between Complete Case and MICE estimates indicates that the results are robust to the missing data mechanism.*
